# Carcinoid Heart Disease and Decreased Overall Survival among Patients with Neuroendocrine Tumors: A Retrospective Multicenter Latin American Cohort Study

**DOI:** 10.3390/jcm8030405

**Published:** 2019-03-23

**Authors:** Deise Uema, Carolina Alves, Marcella Mesquita, Jose Eduardo Nuñez, Timo Siepmann, Martin Angel, Juliana F. M. Rego, Rui Weschenfelder, Duilio R. Rocha Filho, Frederico P. Costa, Milton Barros, Juan M. O’Connor, Ben M. Illigens, Rachel P. Riechelmann

**Affiliations:** 1 Division of Health Care Sciences Center for Clinical Research and Management Education Dresden, Dresden International University, 01067 Dresden, Germany; deiseu@gmail.com (D.U.); timosiepmann.research@gmail.com (T.S.); minwootaurus@gmail.com (B.M.I.); 2 Instituto do Cancer do Estado de Sao Paulo, Sao Paulo 01246-000, Brazil; carolinaacs@hotmail.com (C.A.); cellamesquita@yahoo.com.br (M.M.); ejnunezr@gmail.com (J.E.N.); 3 Department of Clinical Oncology, AC Camargo Cancer Center, Sao Paulo 01509-900, Brazil; miltonb19@gmail.com; 4 Instituto Alexander Fleming, C1426ANZ Buenos Aires, Argentina; martin.angel@hotmail.com; 5 Hospital Universitário Onofre Lopes, Natal 59012-300, Brazil; juliana.oncologia@gmail.com; 6 Hospital Moinhos de Vento, Porto Alegre 90035-001, Brazil; rui.fernando.w@gmail.com; 7 Hospital Universitário Walter Cantídio, Fortaleza 60430-372, Brazil; duilio.rocha@uol.com.br; 8 Hospital Sirio Libanês, São Paulo 01308-050, Brazil; fredericoperegocosta@gmail.com; 9 Hospital de Gastroenterología Bonorino Udaondo, C1264AAA Buenos Aires, Argentina; juanmanuel.oconnor@gmail.com; 10 Department of Neurology, Beth Israel Deaconess Medical Center, Harvard Medical School, Boston, MA 02114, USA

**Keywords:** neuroendocrine tumors, carcinoid heart disease, carcinoid syndrome, somatostatin analogues, metastases

## Abstract

The background to this study was that factors associated with carcinoid heart disease (CHD) and its impacts on overall survival (OS) are scantly investigated in patients (pts) with neuroendocrine tumors (NETs). In terms of materials and methods, a retrospective multicenter cohort study was conducted of factors associated with CHD in advanced NET pts with carcinoid syndrome (CS) and/or elevated urinary 5-hidroxyindole acetic acid (u5HIAA). CHD was defined as at least moderate right valve alterations. The results were the following: Among the 139 subjects included, the majority had a midgut NET (54.2%), 81.3% had CS, and 93% received somatostatin analogues. In a median follow-up of 39 months, 48 (34.5%) pts developed CHD, with a higher frequency in pts treated in public (77.2%) versus private settings (22.9%). In a multivariate logistic regression, unknown primary or colorectal NETs (Odds Ratio (OR) 4.35; *p* = 0.002), at least 50% liver involvement (OR 3.45; *p* = 0.005), and being treated in public settings (OR 4.76; *p* = 0.001) were associated with CHD. In a Cox multivariate regression, bone metastases (Hazard Ratio {HR} 2.8; *p* = 0.031), CHD (HR 2.63; *p* = 0.038), and a resection of the primary tumor (HR 0.33; *p* = 0.026) influenced the risk of death. The conclusions were the following: The incidence of CHD was higher in pts with a high hepatic tumor burden and in those treated in a public system. Delayed diagnosis and limited access to effective therapies negatively affected the lives of NET patients.

## 1. Introduction

Approximately 20–30% of patients with neuroendocrine tumors (NETs) are diagnosed with carcinoid syndrome (CS) in the United States [1], and it is usually associated with liver metastases and reduced overall survival [2,3]. Carcinoid syndrome, characterized by flushing, abdominal cramps, diarrhea, and bronchospasm [1,4,5], is caused by the secretion of vasoactive substances such as serotonin, histamine, prostaglandins, and tachykinins [1,5,6,7,8]. The secretion of these substances, in particular serotonin, can induce tissue fibrosis and lead to complications such as mesenteric, peritoneal, and endocardial fibrosis [8,9]. Fibrotic degeneration of the endocardium causes retraction and fixation of cardiac valves in a combination of regurgitation and stenosis, a condition known as carcinoid heart disease (CHD) [7,10]. When diagnosis is delayed, CHD can culminate with right-sided heart failure [7,10]. About 5–10% of cases have left heart involvement, and in such circumstances, lung carcinoids, patent foramen ovale, or extensive liver metastases should be suspected [11]. Because many patients with CHD do not present with symptoms until cardiopathy is in advanced stages [6,7], international guidelines recommend screening for CHD with an echocardiogram in patients with elevated urinary 5-hidroxyindole acetic acid (u5HIAA) (a metabolite of serotonin) independently of carcinoid syndrome symptoms [9,12].

The exact mechanisms causing CHD are unknown, although chronic exposure to elevated serum levels of serotonin is probably the main causal agent [13]. However, not all NET patients with elevated u5HIAA develop CHD. This observation has led to the investigation of other potential contributing factors for CHD, such as bradykinins, tachykinins, activin A, and tissue growth factor (CTGF), although no definitive marker of CHD has been defined [13]. Clinical factors associated with increased risk of CHD have also been evaluated. Retrospective studies have reported that elevated u5HIAA, the presence of flushing, and prior use of chemotherapy were significantly linked to CHD [14]. In a case-control study of 42 NET patients with elevated urinary 5-HIAA levels conducted by our group, we found that 38% developed CHD in a median follow time of 45.3 months [15]. We also observed that concurrent or prior diagnosis of a cardiovascular comorbid illness (such as coronary insufficiency or arterial hypertension) was associated with an odds ratio of 6.58 (95% confidence interval (CI) 1.09; 39.78; *p* = 0.040) for the presence of CHD. Patients with cardiovascular diseases present with endothelial dysfunction, which could predispose them to CHD in the context of other contributing factors.

Latin America lacks consistent data on cancer statistics and outcomes. Moreover, the structure of the health system, which is divided into public and private healthcare, often leads to a significant disparity in access to cancer treatment, possibly affecting recurrence and survival. While retrospective series have reported that up to 50% of patients with CS can develop carcinoid heart disease (CHD) [4,5,16], data about patients with advanced NETs and CHD in Latin America are lacking. Therefore, we conducted a multicenter study aimed at establishing a collaborative group in order to assess the incidence and risk factors for CHD as well as its impact on patients’ overall survival (OS) in a Latin American cohort.

## 2. Material and Methods

This was a multicenter, retrospective cohort study of consecutive patients treated in eight different hospitals in Latin America (Instituto do Cancer do Estado de Sao Paulo, Sao Paulo, Brazil; Department of Oncology, AC Camargo Cancer Center, Sao Paulo, Brazil; Hospital Sirio Libanês, São Paulo, Brazil; Hospital Moinhos de Vento, Porto Alegre, Brazil; Hospital Universitário Walter Cantídio, Fortaleza, Brazil; Hospital Universitário Onofre Lopes, Natal, Brazil; Hospital de Gastroenterología Bonorino Udaondo, Buenos Aires, Argentina; Instituto Alexander Fleming, Buenos Aires, Argentina). This study was conducted according to ICH GCP guidelines and local laws, and the protocol was submitted and approved by local Ethics Committees. The sample of this study was obtained by the evaluation of all consecutive NET cases in coparticipating hospitals: Each medical chart was reviewed for eligibility. Dubious cases were discussed with other authors so a consensus could be achieved.

All patients included were older than 18 years old, were followed between January 2000 and July 2018, had a diagnosis of advanced NETs (confirmed by biopsy), and had symptoms of carcinoid syndrome (reported as flushing, wheezing, or diarrhea, consistent with NET history) and/or elevated u5HIAA at any moment in the disease history (above the upper normal limit according to local laboratory ranges). At least one transthoracic echocardiogram (TTE) for the evaluation of CHD was required. Because of the retrospective nature of this study, a formal CHD screening protocol was not implemented in each institution. However, it is common practice among the participating centers to screen CHD every one or two years in all patients with elevated u5HIAA or CS, or when guided by symptoms. Demographics, comorbidities, tumor characteristics, oncological treatments, and information about heart conditions were collected. Due to the absence of definitive criteria for the diagnosis of CHD, it was defined in this study as at least moderate right heart valve alterations (valve thickening, reflux/regurgitation, or double lesion–stenosis and regurgitation) visualized by a TTE performed by a professional with years-long experience with CHD.

The coprimary objectives of this study were to evaluate factors that could influence the development of CHD and their impact on OS. Absolute and relative frequencies are summarized in the tables. Continuous variables were evaluated for normal distribution using both histograms and the Shapiro–Wilk test. For continuous variables with a normal distribution, the parametric unpaired Student’s *t*-test was used. When the distribution was non-normal, the nonparametric Mann–Whitney *U* test was used instead. A chi-squared test was used for categorical data.

Factors potentially associated with CHD were included in univariate analysis for CHD incidence, such as gender, age, primary site (foregut (pancreas, stomach, lung), midgut, and others, which included hindgut (colorectal), others, or unknown), time from symptoms until NET diagnosis (in months), the functional status of the tumor, the extent of liver metastases (at least 50% liver involvement, which was classified by investigators based on imaging evaluation), the presence of flushing, cardiovascular comorbidities (defined as any previous or concurrent cardiovascular disease that demanded pharmacological therapy, e.g., coronary insufficiency, cerebral vascular event, or chronic high blood pressure), treatment setting (public vs private), and u5HIAA (mg/24 h) at the time of the first TTE. Variables with *p* < 0.1 were entered into a multivariable logistic regression model for CHD. In terms of factors associated with OS, besides the covariates previously mentioned, we also assessed in a univariate Cox proportional-hazards model the following covariates: The presence of CHD, resection of the primary tumor, and bone metastasis. Covariates deemed as significant (*p* < 0.1) in a univariate regression were then entered into the multivariate Cox proportional-hazards model. For OS, the multivariate stepwise model, which sequentially removes each covariate at a time, was applied until the best OS model was found.

**Figure 1:**
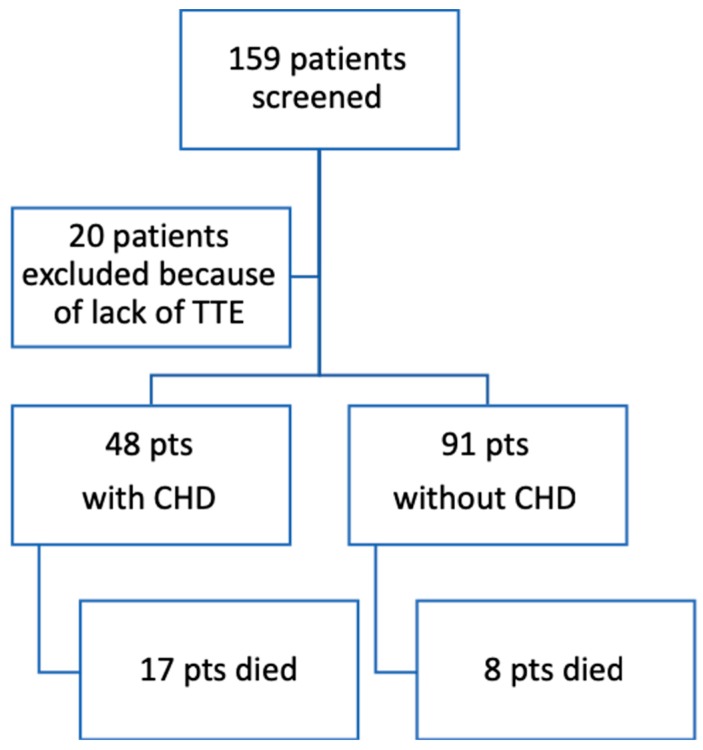
Flowchart of patient selection and inclusion.

All statistical tests were two-sided, with the α level set at 0.05. A Strobe checklist was used to ensure the completeness of the information reported in this retrospective study [17]. All analyses were performed in the whole study population, as patients with significant missing data were excluded (see “Results”, Figure 1).

## 3. Results

One-hundred and fifty-nine patients with advanced disease and carcinoid symptoms and/or elevated u5HIAA were identified in the electronic health records of the participating hospitals. Of these, 139 were eligible and were included in the analysis herein conducted (see flow diagram below).

For CHD incidence, all 139 patients had complete data, while for the OS evaluation, 127 patients out of 139 had all selected factors complete and were included in the analysis. With a median follow-up time of 39 months (range: 2.7–150.6 months), 48 patients (34.5%) developed CHD, and 91 patients (65.5%) remained CHD-free.

The baseline demographic characteristics of the whole population and subgroups based on CHD occurrence are described in Table 1.

Among all patients, midgut tumors were the most common primary site, 81.3% had CS, and 93% received somatostatin analogues.

The mean age at diagnosis of an NET was 56.52 years (±14.8 years) in patients with CHD compared to 51.9 years (±12.54 years) in patients without CHD (*p* = 0.049). Both groups had a similar median time from the beginning of symptoms until diagnosis of an NET (CHD 10.93 months vs non-CHD 9.03 months; *p* = 0.285). A significantly higher proportion of patients with CHD had NETs of other origins, such as colorectal and unknown primary sites (CHD 35.4% vs. non-CHD 11%; *p* = 0.001).

Other factors significantly more frequently encountered among CHD patients were at least 50% of liver volume involved by metastases (CHD 41.7% vs. non-CHD 23.1%; *p* = 0.037), median u5HIAA (mg/24 h) at the time of the first TTE (CHD 40 mg/24 h vs. 18.1 mg/24 h non-CHD (*p* = 0.05)), the proportion of patients treated in a public setting (CHD: Public setting 77.1% vs private setting 22.9%; *p* = 0.001).

Univariate and multivariate logistic regression models showed that primary site, extent of liver metastases, and treatment setting were predictive (*p* < 0.05) for the occurrence of CHD. In multivariate analysis, while holding the two other covariates constant, the odds of presenting with CHD with a primary site of “others” (colorectal, unknown primary, and others such as ovary, peritoneum, lung, etc.) were 4.35 times the odds of presenting with CHD for tumors from the pancreas/stomach or midgut (*p* = 0.002). Similarly, the odds of presenting with CHD with metastases involving more than 50% of liver volume were 3.45 times the odds of presenting with CHD in the group with lower metastatic liver involvement (*p* = 0.005). The odds of presenting with CHD in patients treated in a public setting were 4.76 times the odds of presenting with this condition in patients treated in a private setting (*p* = 0.001), as shown in Table 2.

In terms of OS, the univariate Cox regression showed that age at NET diagnosis, primary site of tumor, occurrence of CHD, resection of primary tumor, setting of treatment (public vs. private), and bone metastases were significantly associated with OS.

In the Cox multivariate regression stepwise model, CHD (HR 2.63, *p* = 0.038), resection of primary NET (HR: 0.33, *p* = 0.026), and bone metastases (HR = 2.8, *p* = 0.031) independently influenced the risk of death, as shown in Table 3.

As shown in Table 4, the most frequently affected heart valve in patients with CHD was the tricuspid valve (50%), followed by a compromise of the tricuspid and pulmonary valves (27.08%) and, to a lesser extent, both right and left heart valves (20.83%). The most common alterations were valve insufficiency (45.8%) and a combination of valve thickening and insufficiency (45.8%). Isolated valve thickening or stenosis was uncommon (20.8%). In terms of severity, the majority of patients had severe alterations in TTEs (31.30%) or severe alterations with dilations of heart chambers or decreased ejection fraction (50%) at the moment of CHD diagnosis. In this sample, only two patients with CHD had corrective valvuloplasty or surgery.

In a median follow-up of 39 months, the median overall survival of patients with CHD was not reached because of the number of events censored. Of the 47 patients with a survival time after CHD diagnosis (time of CHD–months) available, only 17 patients (36.2%) died, while the other 30 patients (63.8%) were still alive at the time of data collection for this study: The mean OS was 68.89 months (95% CI: 50.47–83.32).

After a CHD diagnosis, the OS rate at 1 year was 79%, and at 5 years it was 54%.

Given the strong association between CHD and OS with treatment delivered in the public system, we present in Table 5 the summarized characteristics of patients according to treatment setting. Patients treated in a public setting had a longer time from the beginning of symptoms until a diagnosis of NET, had a higher incidence of CHD, and had more cardiovascular comorbid illnesses. In addition, primary tumors were resected less frequently, and they were less exposed to more than one line of systemic therapy.

## 4. Discussion

In this multicenter retrospective cohort study, the largest conducted in Latin America and among the largest series of CHD cases worldwide, we evaluated the incidence of CHD and the impact on OS of patients with advanced NETs, in addition to the already known adverse prognostic factors. We observed that nearly one-third of patients developed CHD in a median follow-up of 39 months. Factors independently associated with CHD were treatment delivered in a public setting, unknown primary or colorectal NET, and at least 50% liver involvement by metastases. In addition, u5HIAA levels were higher among CHD patients. CHD and bone metastases increased the risk of death, and resection of the primary tumor was a protective factor from mortality.

We found that patients with a larger burden of liver metastases (≥50% of liver volume affected) were more likely to present with CHD [10,11]. CHD is thought to be caused by the action of vasoactive substances in the endocardium [14]. Therefore, it is reasonable to expect that when the production of vasoactive substances exceeds liver metabolism, a larger amount of these substances will reach the right heart, increasing the chances of CHD development [2,6,10,11]. It is believed that some tumors, such as primary NETs in bronchi, ovaries, testes, lymph nodes, and the retroperitoneum, have direct access to systemic circulation, and in this subgroup, CHD frequently occurs in the absence of liver metastases or carcinoid syndrome symptoms [2,3,6,10].

Serotonin seems to play an important role in CHD development, as it induces tissue fibrosis [10,18]. In agreement with previous research, our study showed that urinary serotonin metabolite 5HIAA levels were significantly higher in patients with CHD compared to patients without CHD, supporting the value of u5HIAA as a screening tool for CHD [14,18]. Considering the importance of the hepatic metabolization and location of the primary tumor, it is not surprising that in some studies, u5HIAA has been more precise in predicting the progression of CHD than the radiological progression of the tumor burden [18]. The positive association between cardiovascular comorbidities and CHD observed in our prior study [15] was not found in the present study with a larger sample size. It is possible that the finding from our previous study was a false positive: However, we think that future studies should evaluate cardiovascular comorbid illnesses as a potential risk factor for CHD.

On our opinion, the most important and original result of our study was the significant association between CHD and treatment in a public setting. This likely reflects delayed access or lack of access to effective systemic anticancer therapies and suboptimal supportive care. Therefore, our data strongly suggests that treating carcinoid symptoms, i.e., decreasing exposure to elevated levels of serotonin, prevents or delays CHD. As shown in Table 5, patients in a public setting had a longer interval from the beginning of symptoms until diagnosis of an NET, had lower rates of primary tumor resection, and were less frequently exposed to more than one systemic therapy. The diagnosis of NETs requires a high level of suspicion because of its relative rarity and generic symptoms. Therefore, with a scarcity of ancillary tests, these “indolent tumors” may progress undetected for a prolonged period, leading to a delayed diagnosis. Once the diagnosis is made, NET optimal treatment needs the coordinated action of a multidisciplinary team to ensure the best outcome. In addition to these challenges, in our public healthcare setting, several patients still need to obtain somatostatin analogues via judicialization, as it is not made available in all public services, which delays even further the systemic treatment of metastatic disease. These findings possibly corroborate the prognostic factor “year of diagnosis” found in the Surveillance, Epidemiology and End Results program registry [19]. Having chosen the year of octreotide introduction to the United States (1987), Yao et al. were able to show a positive shift in the survival curve, demonstrating the relevance of the inclusion of somatostatin analogues and enhanced supportive treatment for patients with advanced NETs [16,19,20]. A lack of CHD screening and inappropriate management also seem to play a significant role in the observed higher incidence of CHD in the public system and the related shortened survival.

In our study, CHD was a prognostic factor for mortality, although median survival was not reached due to the low number of events in the median follow-up time of 39 months. Considering that in 1993, Pellikka et al. reported a median overall survival of 1.6 years for CHD patients [4], our median follow-up time seemed appropriate for estimating the median survival of our CHD group. More recent series, such as the study reported by Connolly et al., have reported a 69% OS at 1 year and a 34% OS at 5 years [16], while in our study, the OS rates were 79% at 1 year and 54% at 5 years. These differences in OS rates and median survivals likely reflected our definition of CHD, which considered patients with moderate and severe valve alterations in comparison to other studies that may have included patients with more severe heart valve dysfunctions who were being considered for valve replacements [16]. In addition, a longer follow-up would have been necessary to properly evaluate the OS in our sample [21].

Unfortunately, in contrast to the current evidence that suggests that valve surgery may improve mortality in patients with symptomatic severe right heart valve disease [16,20], only two patients in our sample underwent cardiac surgery, despite more than 80% presenting with severe valve dysfunction as detected by TTE. This likely reflects the poor access to cardiac surgery among patients treated in the public systems of Latin America.

Resection of the primary tumor has also been associated with increased OS and is likely related to a reduction in vasoactive substance production [18] and potentially fatal complications such as bowel obstruction. An alternative explanation for tumor resection and improved OS could be the fact that patients who undergo surgical resection may have a lower metastatic tumor burden and are thus amenable to surgical resection.

The limitations of our study should be pointed out. The retrospective nature of our study may have limited the validity of our findings. We could not evaluate other prognostic markers, such as pro-pro-brain natriuretic peptide, response to treatment, patterns of radiological progression, or even tissue tumor biomarkers, which could have provided us with a deeper understanding of the mechanisms that lead to the development of CHD. The intervals of TTE were not standardized, and this may have underestimated the true incidence of CHD because of the number of patients who were asymptomatic and did not have a recent TTE: TTEs were performed annually or biannually. Although the use of complete case analysis may have favored the observation of patients with better (or worse) prognostic features, the amount of missing data was below 10%. Nonetheless, considering that this was a multicentric study and the largest conducted in a Latin American population, these findings certainly establish the feasibility of such an effort and provide us some guidance for future collaborative prospective studies. It also brings awareness to the alarmingly high incidence of CHD in patients treated in our public systems. Governments should devote more resources to treating NET patients, particularly those with functioning tumors.

In conclusion, in order to diagnose CHD in a timely manner, clinicians should be attentive and aware of the risk of patients with advanced NETs developing such a complication. Our study reinforces the recommendation of performing annual TTEs for patients with elevated u5HIAA. For patients with a low hepatic tumor burden or for those with a primary tumor in the foregut or midgut, screening TTE frequency can possibly be reduced, while for patients with a high hepatic tumor burden and tumors in the hindgut or other locations, as well as those with delayed access or lack of access to antitumor therapies, annual screenings should be strictly maintained or performed even more frequently. Treatment in a public setting was associated with higher chances of developing CHD, underlining the negative impact that disparities in access to healthcare have in terms of cancer outcomes. This highlights the adverse effect of delayed diagnosis and treatment and emphasizes the importance of appropriate CHD screening and early treatment of patients with elevated u5HIAA to offer the best survival chances for patients with advanced NETs.

## Figures and Tables

**Table 1:** Baseline demographic characteristics of the study population.

		With Carcinoid Heart Disease		Without Carcinoid Heart Disease		Whole Population		*p*-Value **
		No. of Subjects	%	No. of Subjects	%	No. of Subjects	%	
Gender	Female	27	56.3	47	52.2	74	53.2	0.735 *
	Male	21	43.7	44	47.8	65	46.8	
Age at diagnosis (years)	Mean ± SD	56.52 ± 14.8		51.9 ± 12.5		53.5 ± 13.5		0.049 ^#^
Time from beginning of symptoms until diagnosis of an NET (months)	Median (range)	10.93 (0–107.9)		7.17 (0–160.6)		9.03 (0–160.6)		0.285
Grouped primary site	Foregut (pancreas/stomach)	5	10.4	13	14.3	18	13.0	0.001 *
	Midgut	26	54.2	68	74.7	94	67.6	
	Colorectal, unknown primary, others	17	35.4	10	11.0	27	19.4	
Staging	Metastatic	45	93.75	78	85.71	123	88.49	
WHO Classification	Grade 1	24	50	34	37.36	58	41.73	
	Grade 2	16	33.33	33	36.26	49	35.25	
	Grade 3	2	4.17	1	1.1	3	2.16	
Median Ki67-% (Range)		2.2 (1–60)		2.0 (1–25)		2.0 (1–60)		
Differentiation	Well differentiated	42	87.5	82	90.11	124	89.21	
Functional status	Functioning	42	87.5	71	78.0	113	81.3	0.257 *
Flushing	Yes	31	64.6	48	52.7	79	56.8	0.246 *
Liver metastases	At least 50% involvement	20	41.7	21	23.1	41	29.5	
Bone metastases	Yes	8	16.7	11	12.09	19	13.67	
Cardiovascular comorbidities	Yes	20	41.7	40	44.0	60	43.2	0.937 *
Treatment setting	Public	37	77.1	42	46.2	79	56.8	0.001 *
	Private	11	22.9	49	53.8	60	43.2	
Resection of primary tumor	Yes	24	51.0	61	67.0	85	61.6	
Metastasectomy	Yes	14	29.2	33	36.3	47	33.8	
u5HIAA (mg/24 h) at 1st TTE	Median (Range)	40 (5.4–271.2)		18.1 (2.8–22365)		23.7 (2.8–22365)		0.05 ^##^

** *p*-value presented for comparison of covariates between patients with carcinoid heart disease (CHD) and without CHD; * chi-squared test; ^#^ parametric Student’s *t*-test; ^##^ nonparametric Mann–Whitney *U* test; SD = standard deviation; NET = neuroendocrine tumor; u5HIAA = urinary 5-hydroxyindole-3-acetic acid; TTE = transthoracic echocardiogram.

**Table 2:** Results of univariate and multivariate analyses for CHD incidence. CI: Confidence interval; OR: Odds Ratio.

Covariates	Univariate OR (CI 95%)	Univariate *p*-Value	Multivariate OR (CI 95%)	Multivariate *p*-Value
Age at diagnosis	1.03 (1.00; 1.05)	0.057		
Gender Female	1.20 (0.60; 2.43)	0.605		
Primary site Hindgut, unknown primary, or others	2.63 (1.72; 4.00)	<0.001	4.35 (1.67; 11.11)	0.002
Time from symptoms until NET diagnosis (months)	1.00 (0.99; 1.02)	0.616		
Functioning tumor	1.97 (0.73; 5.30)	0.178		
More than 50% liver involvement	2.5 (1.61; 3.85)	<0.001	3.45 (1.47; 8.33)	0.005
Treatment in public setting	4.55 (2.33; 8.33)	<0.001	4.76 (1.92; 11.11)	0.001
Presence of flushing	1.63 (0.79; 3.36)	0.182		
Cardiovascular comorbidities	0.91 (0.45; 1.85)	0.796		
u5HIAA at 1st TTE	1 (0.999; 1)	0.657		

**Table 3:** Results of univariate Cox regression and multi-Cox regression for overall survival (OS).

Covariates	Univariate OR (CI 95%)	Univariate *p*-Value	Multivariate * OR (CI95%)	Multivariate * *p*-Value
Age at diagnosis	1.06 (1.02; 1.11)	0.001	1.05 (1.00; 1.09)	0.028
Gender Female	1.78 (0.75; 4.26)	0.193		
Primary site Hindgut, unknown primary, or others	2.70 (1.12; 6.67)	0.026		
CHD	3.75 (1.53; 9.20)	0.004	2.63 (1.05; 6.56)	0.038
Primary tumor resection	0.27 (0.11; 0.65)	0.004	0.33 (0.13; 0.87)	0.026
Functioning tumor	3.51 (0.47; 26.16)	0.221		
More than 50% liver involvement	1.28 (0.58; 3.03)	0.579		
Treatment in public setting	4.00 (1.22; 11.11)	0.013		
Bone metastases	2.7 (1.10; 6.62)	0.030	2.80 (1.10; 7.13)	0.031

* Multivariate stepwise model.

**Table 4:** Echocardiographic alterations in patients with CHD.

**Heart valves affected**	***N***	**%**
Tricuspid	24	50.00
Tricuspid and pulmonary	13	27.08
Right and left heart	10	20.83
**Type of alteration**	***N***	**%**
Valve thickening	1	2.08
Valve insufficiency	22	45.80
Thickening and insufficiency	22	45.80
Stenosis	2	4.17
**Severity**	***N***	**%**
Mild	2	4.17
Moderate	7	14.60
Severe	15	31.30
Severe with dilation or decreased ejection fraction	24	50.00

**Table 5:** Summarized characteristics of patients according to treatment setting (pts = patients).

Covariates	Public	Private
Median age at diagnosis	58 years	49 years
Time from beginning of symptoms until NET diagnosis	12 months	6.7 months
CHD	46.8% (37/79 pts)	18.3% (11/60 pts)
More than 50% liver involvement	25.3% (20/79 pts)	35% (21/60 pts)
Cardiovascular comorbid illnesses	48.1% (38/79 pts)	36.6% (22/60 pts)
Primary tumor resected	54.4% (43/79 pts)	70% (42/60 pts)
Bone metastases	8.8% (7/79 pts)	20% (12/60 pts)
Flushing	65.8% (52/79 pts)	45% (27/60 pts)
Carcinoid syndrome	87.3% (69/79 pts)	73.3% (44/60 pts)
Somatostatin analogues use	91.1% (72/79 pts)	95% (57/60 pts)
Received more than one systemic treatment	27.8% (22/79 pts)	60% (36/60 pts)

## References

[1] Raja S.G., Bhattacharyya S., Davar J., Dreyfus G.D. (2010). Surgery for carcinoid heart disease: Current outcomes, concerns and controversies. Future Cardiol..

[2] Feldman J.M., Jones R.S. (1982). Carcinoid Syndrome from Gastrointestinal Carcinoids without Liver Metastasis. Ann Surg.

[3] Haq A.U., Yook C.R., Hiremath V., Kasimis B.S. (1992). Carcinoid Syndrome in the Absence of Liver Metastasis: A Case Report and Review of Literature. Med. Pediatr. Oncol..

[4] Pellikka P.A., Tajik A.J., Khandheria B.K., Seward J.B., Callahan J.A., Pitot H.C., Kvols L.K. (1993). Carcinoid heart disease. Clinical and echocardiographic spectrum in 74 patients. Circulation.

[5] Connolly H.M., Schaff H.V., Mullany C.J., Rubin J., Abel M.D., Pellikka P.A. (2001). Surgical Management of Left-Sided Carcinoid Heart Disease. Circulation.

[6] Grozinsky-Glasberg S., Grossman A.B., Gross D.J. (2015). Carcinoid Heart Disease: From Pathophysiology to Treatment—“Something in the Way It Moves”. Neuroendocrinology.

[7] Dobson R., Burgess M.I., Pritchard D.M., Cuthbertson D.J. (2014). The clinical presentation and management of carcinoid heart disease. Int. J. Cardiol..

[8] Mota J.M., Sousa L.G., Riechelmann R.P. (2016). Complications from carcinoid syndrome: Review of the current evidence. Ecancermedicalscience.

[9] Pape U.-F., Perren A., Niederle B., Gross D., Gress T., Costa F., Arnold R., Denecke T., Plöckinger U., Salazar R. (2012). ENETS Consensus Guidelines for the Management of Patients with Neuroendocrine Neoplasms from the Jejuno-Ileum and the Appendix Including Goblet Cell Carcinomas. Neuroendocrinology.

[10] Gustafsson B.I., Hauso O., Drozdov I., Kidd M., Modlin I.M. (2008). Carcinoid heart disease. Int. J. Cardiol..

[11] Fox D.J., Khattar R.S. (2004). Carcinoid Heart Disease: Presentation, Diagnosis, and Management. Heart.

[12] Riechelmann R.P., Weschenfelder R.F., Costa F.P., Chaves Andrade A., Bersch Osvald A., Quidute A.R.P., Dos Santos A., Hoff A.A.O., Gumz B., Buchpiguel C. (2017). Guidelines for the management of neuroendocrine tumours by the Brazilian gastrointestinal tumour group. Ecancermedicalscience.

[13] Ferrari A., Glasberg J., Riechelmann R. (2018). Carcinoid syndrome: Update on the pathophysiology and treatment. Clinics.

[14] Møller J.E., Connolly H.M., Rubin J., Seward J.B., Modesto K., Pellikka P.A. (2003). Factors Associated with Progression of Carcinoid Heart Disease. N. Engl. J. Med..

[15] Alves C., Mesquita M., Silva C., Soeiro M., Hajjar L., Riechelmann R.P. (2018). High tumour burden, delayed diagnosis and history of cardiovascular disease may be associated with carcinoid heart disease. Ecancermedicalscience.

[16] Connolly H.M., Schaff H.V., Abel M.D., Rubin J., Askew J.W., Li Z., Inda J.J., Luis S.A., Nishimura R.A., Pellikka P.A. (2015). Early and late outcomes of surgical treatment in carcinoid heart disease. J. Am. Coll. Cardiol..

[17] STROBE (2007). STROBE 2007 (v4) Statement—Checklist of items that should be included in reports of cohort studies. PLoS Med..

[18] Dobson R., Burgess M.I., Valle J.W., Pritchard D.M., Vora J., Wong C., Chadwick C., Keevi B., Adaway J., Hofmann U. (2014). Serial surveillance of carcinoid heart disease: Factors associated with echocardiographic progression and mortality. Br. J. Cancer.

[19] Yao J.C., Hassan M., Phan A., Dagohoy C., Leary C., Mares J.E., Abdalla E.K., Fleming J.B., Vauthey J.N., Rashid A. (2008). One hundred years after “carcinoid”: Epidemiology of and prognostic factors for neuroendocrine tumors in 35,825 cases in the United States. J. Clin. Oncol..

[20] Warner R.R.P., Castillo J.G. (2015). Carcinoid heart disease: The challenge of the unknown known. J. Am. Coll. Cardiol..

[21] Westberg G., Wängberg B., Ahlman H., Bergh C.H., Beckman-Suurküla M., Caidahl K. (2001). Prediction of prognosis by echocardiography in patients with midgut carcinoid syndrome. Br. J. Surg..

